# Effects of Vitamin D3 on the NADPH Oxidase and Matrix Metalloproteinase 9 in an Animal Model of Global Cerebral Ischemia

**DOI:** 10.1155/2018/3273654

**Published:** 2018-04-18

**Authors:** Milica Velimirović, Gordana Jevtić Dožudić, Vesna Selaković, Tihomir Stojković, Nela Puškaš, Ivan Zaletel, Milica Živković, Vesna Dragutinović, Tatjana Nikolić, Ankica Jelenković, Djordje Djorović, Aleksandar Mirčić, Nataša D. Petronijević

**Affiliations:** ^1^Institute of Clinical and Medical Biochemistry, School of Medicine, University of Belgrade, Pasterova 2, Belgrade, Serbia; ^2^Military Medical Academy, Crnotravska 17, Belgrade, Serbia; ^3^Institute of Histology and Embryology “Aleksandar Đ. Kostić”, School of Medicine, University of Belgrade, Pasterova 2, Belgrade, Serbia; ^4^Institute of Medical Chemistry, School of Medicine, University of Belgrade, Belgrade, Serbia; ^5^Institute for Biological Research “Siniša Stanković”, University of Belgrade, Belgrade, Serbia; ^6^Institute of Anatomy “Niko Miljanić”, School of Medicine, University of Belgrade, Dr Subotića 4, Belgrade, Serbia

## Abstract

Decreased blood flow in the brain leads to a rapid increase in reactive oxygen species (ROS). NADPH oxidase (NOX) is an enzyme family that has the physiological function to produce ROS. NOX2 and NOX4 overexpression is associated with aggravated ischemic injury, while NOX2/4-deficient mice had reduced stroke size. Dysregulation of matrix metalloproteinases (MMPs) contributes to tissue damage. The active form of vitamin D3 expresses neuroprotective, immunomodulatory, and anti-inflammatory effects in the CNS. The present study examines the effects of the vitamin D3 pretreatment on the oxidative stress parameters and the expression of NOX subunits, MMP9, microglial marker Iba1, and vitamin D receptor (VDR), in the cortex and hippocampus of Mongolian gerbils subjected to ten minutes of global cerebral ischemia, followed by 24 hours of reperfusion. The ischemia/reperfusion procedure has induced oxidative stress, changes in the expression of NOX2 subunits and MMP9 in the brain, and increased MMP9 activity in the serum of experimental animals. Pretreatment with vitamin D3 was especially effective on NOX2 subunits, MMP9, and the level of malondialdehyde and superoxide anion. These results outline the significance of the NOX and MMP9 investigation in brain ischemia and the importance of adequate vitamin D supplementation in ameliorating the injury caused by I/R.

## 1. Introduction

Cerebral ischemia is caused by various disorders, such as myocardial infarction, stroke, or peripheral vascular disease [1]. These diseases are the most common causes of mortality and morbidity worldwide and represent a significant social and economic burden [2, 3].

Decreased blood flow disturbs homeostasis of the brain cells and causes noncontrolled formation of reactive oxygen species (ROS). ROS can be derived from different sources, including mitochondria, xanthine oxidase, uncoupled nitric oxide synthase (NOS), and cyclooxygenase [4]. All of these enzymes produce ROS as a by-product. However, recently the enzyme family of NADPH oxidase (NOX), with the single known function of producing ROS, has been recognized as an important source of free radicals in the ischemic brain [4]. The NOX enzymes play essential roles in many physiological processes including functioning of the brain, immune system, vasculature, and signal transduction [4, 5]. Seven transmembrane NOX family members (NOX1–5 and Duox1/2) transport electrons across biological membranes to reduce oxygen to superoxide anion (O_2_^·−^). NOX2 is the most widely distributed among NOX isoforms, and it is composed of two membrane subunits (gp91^phox^ (referred as NOX2) and gp22^phox^) and four cytosolic subunits (p47^phox^, gp67^phox^, gp40^phox^, and Rac) [5]. NOX2 expression is present in the different cells in the central nervous system (CNS) including microglia [6], neurons [7, 8], and astrocytes [9]. Besides NOX2, the attention has recently been brought to NOX4 that represents the most common isoform in the vascular network, especially in the central nervous system [10]. NOX4 shares 39% similarity with NOX2 and requires only p22 subunits [11] for ROS production. The expression of the NOX is changed in the neurodegenerative diseases, aging, seizures, and ischemic stroke [11]. To date, NOX2 and NOX4 isoforms are the most implicated to contribute to ROS generation and pathology following cerebral ischemia [5].

An increase in NOX2 activity and expression has repeatedly been found in different rodent models of stroke [5, 11, 12]. The overexpression of NOX2 and NOX4 after stroke has been associated with aggravated ischemic injury, and the stroke size was reduced in NOX2- and NOX4-deficient mice [4, 13].

Investigations in patients have also demonstrated involvement of NOX in stroke pathophysiology [14] although the patient sample size was small. Hence, these NOX isoforms represent a potential therapeutic target. Many NOX inhibitors have been proposed; however, the ideal one has not been found so far due to the lack of selectivity, favorable toxicity, and bioavailability [15, 16].

Cerebral ischemia also leads to deregulation of matrix metalloproteinases 2 and 9 (MMP) [17] causing matrix degradation, blood-brain barrier (BBB) disruption, hemorrhage, and brain edema [18, 19]. Park et al. [20] have shown that the activity of MMP9 in serum reflects the activity of MMP9 in the brain. In acute stroke, higher serum MMP9 levels are found to correlate with infarct volume, disease severity, late hemorrhage, mortality, and major disability [21–24]. However, MMP-targeted inhibitors that are not strictly specific showed insufficient clinical benefits [25, 26]. Findings that MMP9 activity and expression correspond to the increased expression of NOX2 [27] offer the opportunity to regulate MMP9 by inhibiting NOX2.

In ischemic tissue, reperfusion is of utmost importance but reintroducing of oxygen leads to even greater ROS production and mitochondrial dysfunction [9]. The beginning of the reperfusion period is a perfect time to inhibit ROS production. Although the right inhibitors have not been synthesized yet, adjuvant nutritional interventions following brain ischemia or trauma that are safe, low cost, and accessible have given promising results [28–30]. Besides applying vitamin treatment after brain ischemia, several studies have demonstrated that vitamin supplementation of the subjects with increased risk for brain ischemia could be beneficial [31, 32]. Vitamin D3 is currently the focus of investigation since it is effective in neuroprotection and immunomodulation and in decreasing inflammation in the CNS [33–35]. Vitamin D3 exhibits effects in the body through the vitamin D nuclear receptors (VDR) that are present in the brain both in neurons and in glial cells [36, 37]. Inverse associations between the serum levels of 25-hydroxyvitamin D3 and the risk of hypertension, cerebrovascular, and cardiovascular incidents have been demonstrated [31, 38]. Many observational studies are in favor of the hypotheses that vitamin D3 supplementation could be protective against stroke [31]. However, only a few interventional studies are available, and their results differ, probably due to methodological limitations [31, 32]. Problems in these trials have included the dosage of vitamin D3, individual variations in response to vitamin D3 supplementation, and expression of the results as an incidence of cardiovascular or cerebrovascular events instead of addressing the severity of the outcome.

This study was designed to assess the effects of vitamin D3 pretreatment on the expression of neuronal marker NeuN, subunits of NADPH oxidase, MMP9, and microglial marker (Iba1), as well as on the oxidative stress parameters including the levels of O_2_^·−^, malondialdehyde (MDA), and reduced glutathione (GSH) and the activities of glutathione peroxidase (GPx), glutathione reductase (GR), superoxide dismutase (SOD), and gamma-glutamylcysteine ligase (γGCL) in the cortex and hippocampus of Mongolian gerbils subjected to global cerebral ischemia followed by 24 hours of reperfusion (I/R). The activity of MMP9 was measured in the serum. The expression of VDR, as an indicator of vitamin D3 activity, was also measured in the investigated brain structures.

## 2. Method

### 2.1. Animals

Adult 8-week-old male Mongolian gerbils were used in this study. The animals were housed individually in wire hanging cages located within a temperature-controlled animal vivarium maintained under 12 : 12 h light/dark schedule. Food and water were available ad libitum. Animals were treated in accordance with the NIH Guide for Care and Use of Laboratory Animals (1985) and European Communities Council Directive (86/609/EEC). All efforts were made to minimize animal suffering and to reduce the number of animals used.

### 2.2. Global Cerebral Ischemia

The gerbils were randomly divided into three groups. Group I (*n* = 20) was sham-operated animals, which underwent the same surgical procedure without vessel occlusion (control), group II (*n* = 20) was subjected to ischemia/reperfusion (I/R), and group III (*n* = 20) was treated with vitamin D3 and then subjected to I/R (VitD + I/R). Ischemia was provoked by bilateral common carotid occlusion (BCAO) for 10 minutes. At the end of the 10-minute occlusion period, the clips were removed and the cerebral blood flow was recovered. Reperfusion lasted for 24 hours, after which gerbils were dispatched by decapitation. The procedure of BCAO and reperfusion is done in accordance with Selakovic et al. [39]. 1,25-(OH)_2_ vitamin D3 (Calcitriol, Abbott lab) was injected intraperitoneally (i.p.) at a dose of 1 *μ*g/kg/day [40], during the 7 days prior to ischemia. Sham-operated and I/R-subjected animals were treated with i.p. injections of saline (NaCl 0.9%) in the same time points with corresponding volumes.

### 2.3. Quantitative Western Blot Analysis

For Western blot analysis, animals were sacrificed by rapid cervical dislocation and decapitation without anesthesia. The brains were removed, and the dorsolateral frontal cortex and hippocampus were dissected. These brain regions were homogenized in lysis buffer (50 mM Tris-HCl (pH 7.4), 150 mM NaCl, 1% IGEPAL CA-630, 1 mM phenylmethylsulphonyl fluoride (Sigma-Aldrich, P7626), protease inhibitor cocktail (Sigma-Aldrich, P8340), 200 mM sodium orthovanadate (Sigma, Germany), and 1 M sodium fluoride (Merck, USA)) on ice for 30 min, followed by centrifugation (18,000*g* for 15 min at 4°C), and the supernatants were collected. Protein concentrations were determined by the method of Bradford using bovine serum albumin as a standard (Sigma). Equal amounts of protein (50 *μ*g) from each sample were separated by SDS-PAGE on 10% and 12% gels and transferred to nitrocellulose membranes (Bio-Rad, Hercules, CA). Membranes were blocked at room temperature for 1 hour in 5% nonfat dry milk in Tris-buffered saline/0.1% Tween 20 (TBST). The following primary antibodies were used in this study: mouse polyclonal NeuN (1 : 1000, Millipore, Germany), mouse polyclonal 91-^phox^ (1 : 500, Santa Cruz, CA), rabbit polyclonal p22-^phox^ (1 : 500, Santa Cruz, CA), rabbit polyclonal p47-^phox^ (1 : 500, Santa Cruz, CA), rabbit polyclonal p67-^phox^ (1 : 500, Santa Cruz, CA), rabbit monoclonal NOX4 (1 : 5000, Abcam, UK), rabbit polyclonal VDR (1 : 1000, Abcam, UK), goat polyclonal anti-Iba1 (1 : 250, Abcam, UK), and rabbit polyclonal anti-MMP9 (1 : 1000, Sigma-Aldrich, Germany). After incubation with primary antibodies, the membranes were incubated with the HRP-labeled secondary anti-goat antibody (1 : 2000, Southern Biotech, USA), anti-rabbit antibody (1 : 2000, Southern Biotech, USA), or anti-mouse antibody (1 : 2000, Southern Biotech, USA) in TBST for 1 hour at room temperature. Five subsequent washes with 0.1% TBST were performed between each step. All membranes were stripped and reprobed with anti-actin antibodies (1 : 10000, mouse monoclonal, Sigma, USA). The signal was detected by enhanced chemiluminescence and subsequent exposure on an X-ray film. Western blot was scanned, and densitometric analysis was performed using ImageQuant 5.2.

### 2.4. Immunohistochemistry

Brains were fixated in 4% formaldehyde solution for 24 h at 4°C followed by sucrose infiltration for 2 days at 4°C (30% sucrose in 0.1 M phosphate buffer), frozen by immersion in 2-methyl-butane (Sigma-Aldrich, USA), and precooled and stored at −80°C until cutting. Serial transverse sections (30 *μ*m thick) were cut on a cryostat (Leica Instruments, Nußloch, Germany). Brain sections were rehydrated and treated with citrate buffer (pH 6.0) in the microwave for antigen retrieval. Endogenous peroxidase activity was blocked with 3% H_2_O_2_, and nonspecific labeling was blocked by a commercial protein block (Vector, UK). Slices were incubated with primary antibodies—mouse polyclonal NeuN (1 : 1000, Millipore, Germany), rabbit monoclonal gp91^phox^ (1 : 100, Abcam, UK), rabbit monoclonal p22^phox^ (1 : 300, Abcam), and goat polyclonal anti-Iba1 (1 : 250, Abcam, UK)—overnight at room temperature. Labeling was performed using a biotin-conjugated secondary antibody, followed by streptavidin-HRP, and visualization was done with 3,3′-diaminobenzidine (DAB) chromogen (all components from the Vectastain ABC HRP Kit, PK-4002, Vector, UK). Finally, the sections were dehydrated and mounted with DPX (Sigma-Aldrich). Pictures were taken using an Olympus BX41 microscope with an Olympus C5060-ADU wide-zoom digital camera. NeuN-positive cells were manually quantified using ImageJ software. We have counted cell from each side, left and right, in both structures. From each animal, six randomly selected sections of the cortex and hippocampus per side were considered for counting at 400x magnification.

### 2.5. Gel Zymography

Serums from all experimental groups were collected during decapitation. For gelatin zymography, serums were diluted in 200 g/L sucrose to prepare the samples as described in Dragutinović et al. [41]. Equal amounts of proteins were separated in nonreducing conditions on 7.5% SDS-PAGE gel copolymerized with 0.01 g/L gelatin. Gels were washed for 45 min in 2.5% Triton X-100 to remove SDS and incubated for 60 hours in 0.1 mol/L glycine, 50 mmol/L Tris-HCl, 5 mmol/L CaCl_2_, 1 *μ*mol/L ZnCl_2_, and 0.5 mol/L NaCl (pH 8.3) at 37°C. Gels were then stained with 0.1% Coomassie blue G-250 for 2 hours and distained with 5% acetic acid until white proteolytic bands appeared on the blue background. Wet zymograms were digitized using a scanner, and gelatinolytic activity was quantified with ImageQuant 5.2.

### 2.6. Brain Preparation for Biochemical Measurements

The dorsolateral frontal cortex and hippocampus were dissected, and the crude synaptosomal fraction was prepared according to the method of Whittaker and Barker [42]. Using a spectrophotometric assay, reduced glutathione content [43], MDA content [44], superoxide anion (O_2_^·−^) levels [45], activity of antioxidative enzymes (superoxide dismutase (SOD), glutathione peroxidase (GPx), and glutathione reductase (GR)) [46–48], *γ*GCL activity [49], and protein concentration [50] were measured.

### 2.7. Transmission Electron Microscopy (TEM)

For transmission electron microscopy, fixed brain samples were sliced using a microslicer and fixed in 3% solution of glutaraldehyde (Merck, Darmstadt, Germany) in 0.1 M cacodylate buffer (pH 7.4) (Merck, Darmstadt, Germany) overnight at +40°C. Following osmification, pellets were dehydrated in an ethanol gradient (Galenikaad, Serbia), cleared in propylene oxide (Merck, Germany), and then embedded in Epon (Merck, Germany). Semithin sections were cut using a diamond knife on a Leica Ultracut UCT EM FCS ultramicrotome (Leica Microsystems, Austria), stained with toluidine blue, and analyzed by an Olympus BX41 light microscope (Olympus, Japan). All the slides were photodocumented with an Olympus C-5060 ADU wide-zoom camera and the Olympus DP-soft Image Analyzer program (Olympus GmbH, Germany). The ultrathin sections from chosen representative areas were cut with the same ultramicrotome, collected on copper grids, and stained with uranyl acetate and lead citrate (SERVA Electrophoresis, Germany). The sections were examined by FEI Morgagni 268D transmission electron microscopy (FEI Europe, Netherlands) and photographed with a Mega View III Soft Imaging System digital camera (Olympus Soft Imaging Solutions, Germany).

### 2.8. Statistical Analysis

The Kolmogorov-Smirnov test has been used to determine normality of the distribution of the numerical values, and Levene's test to test equality of variances between samples. The results are expressed as the mean ± standard error (SE) and were analyzed using the one-way ANOVA with the Fisher post hoc test. The differences were considered to be significant at values of *p* < 0.05.

## 3. Results

### 3.1. I/R Procedure and Vitamin D3 Pretreatment Did Not Change the Expression of NeuN, but Transmission Electron Microscopy Revealed Subtle Subcellular Changes

**Figure 1:**
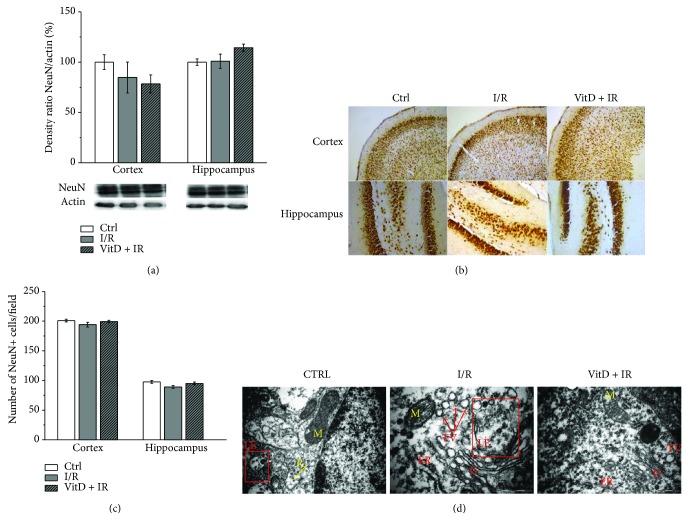
(a) Expression of NeuN. Group I (*n* = 6) was sham-operated animals, which underwent the same surgical procedure without vessel occlusion (control), group II (*n* = 6) was subjected to ischemia/reperfusion (I/R), and group III (*n* = 5) was treated with vitamin D3 and then subjected to I/R (VitD + I/R). All experiments were performed in synaptosomal fraction in different brain structures. (b) Immunohistochemical staining of NeuN-positive cells in gerbil brain slices. Magnification of the upper and lower images is 100x and 200x, respectively. (c) Number of cells per field in group I (sham-operated animals), group II (subjected to ischemia/reperfusion (I/R)), and group III (treated with vitamin D3 and then subjected to I/R (VitD + I/R)). (d) The TEM analysis of neurons. (Left) The neuronal cell from the control group shows late endosomes (LE) with an intact membrane, ribosomes (R), and mitochondria (M) (bar = 1 *μ*m). (Middle) The neuron after I/R shows late endosome with membrane rupture, accumulation of transport vesicles (TV), and more ribosomes (bar = 0.5 *μ*m). (Right) The neuron cell after the VitD + I/R procedure shows intact endosome, some vesicles, Golgi apparatus (G), and rough endoplasmic reticulum (ER) (bar = 1 *μ*m).

Western blot analyses showed unchanged expression of neuronal marker (NeuN) among groups in both structures (Figure 1(a)). Immunohistochemistry has confirmed these results. Numerous NeuN-positive cells were found both in the cortex and in the hippocampus (Figure 1(b)), and quantification of NeuN-positive cells has shown the absence of a significant difference in cell number among experimental groups in both structures (Figure 1(c)). However, transmission electron microscopy (Figure 1(d)) revealed subtle subcellular changes in the form of accumulation of transport vesicles and rupture of the late endosome membrane in the cells of animals subjected to I/R. These changes were less prominent in the brain of the gerbils treated with vitamin D3 before I/R.

### 3.2. Vitamin D3 Pretreatment Has Reversed Changes of the Expression of Membrane NADPH Oxidase Subunits Caused by I/R

**Figure 2:**
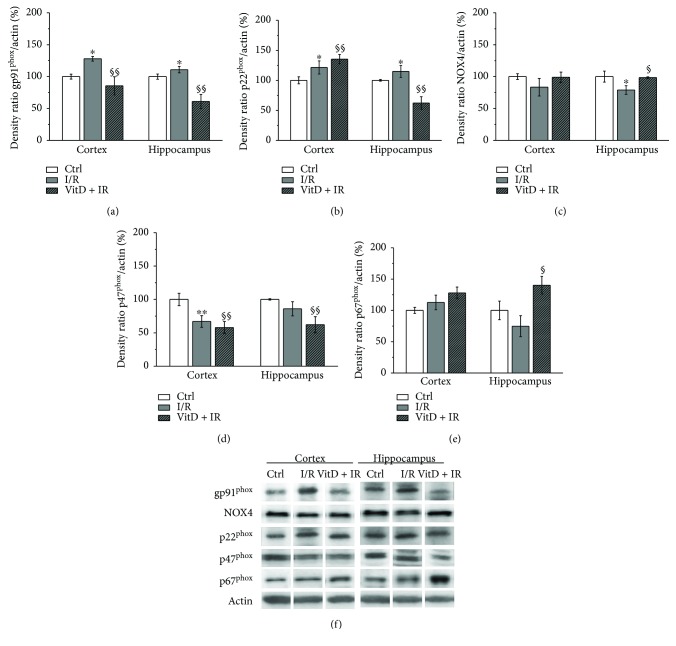
Expression of NADPH oxidase subunits: (a) gp91^phox^, (b) p22^phox^, (c) NOX4, (d) p47phox, and (e) p67^phox^. Group I (*n* = 6) was sham-operated animals, which underwent the same surgical procedure without vessel occlusion (control), group II (*n* = 6) was subjected to ischemia/reperfusion (I/R), and group III (*n* = 5) was treated with vitamin D3 and then subjected to I/R (VitD + I/R). All experiments were performed in synaptosomal fraction in different brain structures. Results are presented as mean ± SE. ^∗^*p* < 0.05 versus control, ^∗∗^*p* < 0.01 versus control, ^§^*p* < 0.05 versus I/R, and ^§§^*p* < 0.01 versus I/R.

The I/R procedure was followed by increased expression of gp91^phox^ (Figure 2(a)) and p22^phox^ (Figure 2(b)) in both investigated brain structures. The expression of p47^phox^ was decreased (Figure 2(d)) in the cortex while the expression of NOX4 (Figure 2(c)) was decreased in the hippocampus with respect to the control group. I/R did not cause any change in the expression of p67^phox^ (Figure 2(e)). Supplementation with vitamin D3 prior to I/R has significantly decreased expression of gp91^phox^ and p47^phox^ in the cortex and hippocampus with respect to I/R, and in the hippocampus, expression of these subunits was also significantly decreased with respect to controls. Furthermore, in the VitD + I/R group, expression of p22^phox^ in the hippocampus was decreased with respect to I/R and controls, while the expression of p22^phox^ in the cortex and the expression of p67^phox^ in the hippocampus were increased with respect to the I/R group and controls. Vitamin D3 caused normalization of NOX4 expression in the hippocampus.

**Figure 3:**
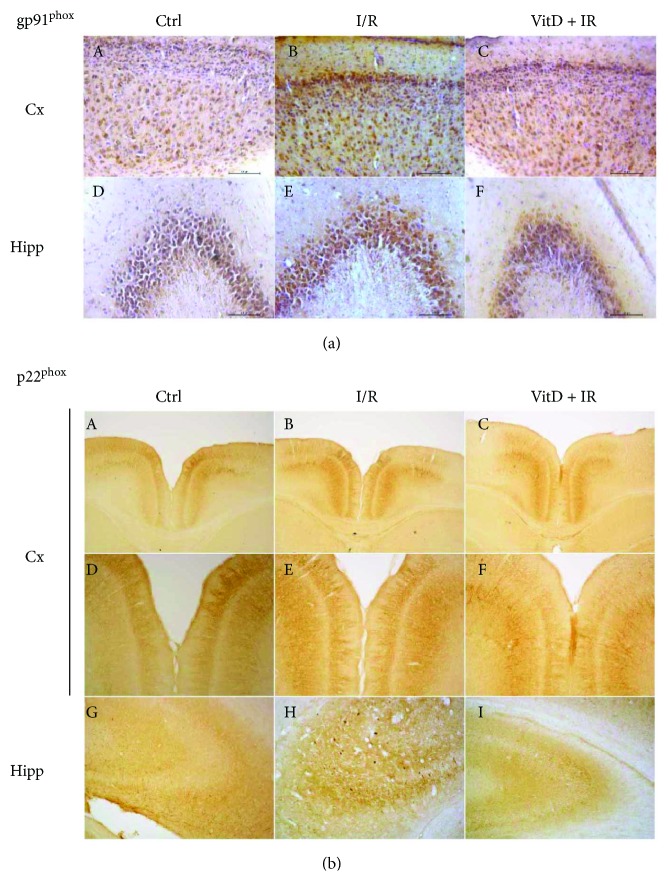
Immunohistochemical staining of gp91^phox^ and p22^phox^ in gerbil brain slices. Magnification of images for (a) from A–F is 200×, for (b) from A–C is 40×, and for (b) from D–I is 100×.

Immunohistochemical staining of gp91^phox^ in regions of the retrosplenial agranular (RSA) cortex and retrosplenial granular (RSG) cortex showed gp91^phox^-positive cells in all cortex layers in all investigated groups. In control and VitD + I/R groups (Figures 3(a), A and 3(a), C), the cell membrane reacted to staining, with a weaker reaction in the cytoplasm. In the I/R group (Figure 3(a), B), cells showed an extensively colored membrane and a strong reaction in the cytoplasm. Gp91^phox^-positive cells were also found in the hippocampus (CA1, CA2/3, and dentate gyrus (DG)). As in the cortex, the stronger staining was noticed in the I/R group (Figure 3(a), E) compared to control and VitD + I/R groups (Figures 3(a), D and 3(a), F).

Immunohistochemical staining on p22^phox^ in regions of the RSA cortex and RSG cortex in all experimental groups revealed a regular, dense network mainly composed of parallel-arranged apical dendrites of pyramidal neurons, as well as of some impregnated pyramidal perikarya. Immunoreactivity was stronger in the I/R group (Figures 3(b), B and 3(b), E) and even more prominent in the VitD + I/R group (Figures 3(b), C and 3(b), F). Interestingly, the CA2/3 hippocampal region responded to experimental conditions more than the CA1 and DG regions. In the control group, a network of long impregnated dendrites and sporadic perikarya can be seen (Figure 3(b), G). Numerous and prominent stainings of cell bodies are observed in the I/R group (Figure 3(b), H). Vitamin D3 pretreatment decreased immunoreactivity with respect to the control group (Figure 3(b), I).

### 3.3. Expression of Iba1 Was Unchanged in All Experimental Groups

**Figure 4:**
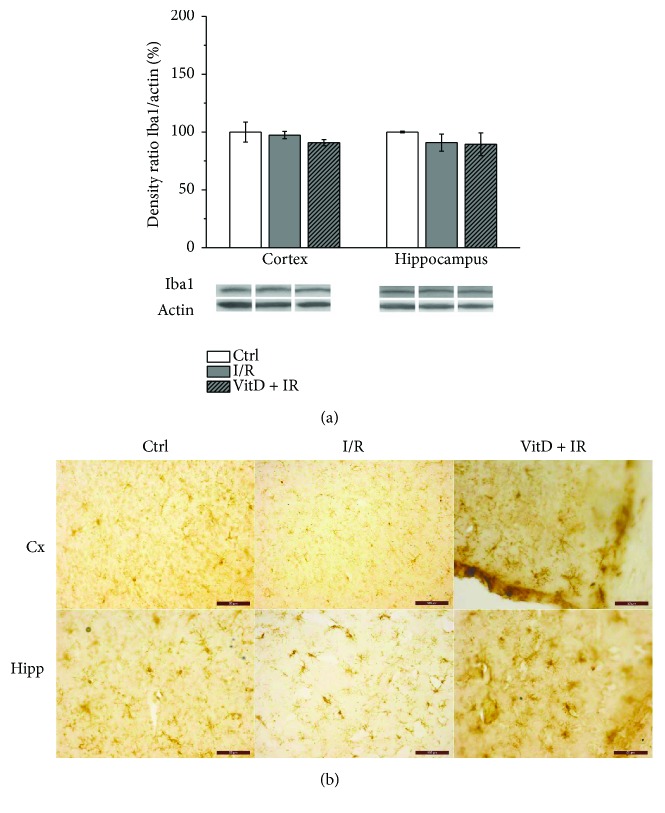
(a) Expression of Iba1. Group I (*n* = 6) was sham-operated animals, which underwent the same surgical procedure without vessel occlusion (control), group II (*n* = 6) was subjected to ischemia/reperfusion (I/R), and group III (*n* = 5) was treated with vitamin D3 and then subjected to I/R (VitD + I/R). All experiments were performed in synaptosomal fraction in different brain structures. Results are presented as mean ± SE. (b) Immunohistochemical staining of Iba1-positive cells in gerbil brain slices. Magnification of images is 200x.

Expression of Iba1 (Figure 4(a)) was unchanged in the cortex and hippocampus in experimental groups compared to control. Immunohistochemistry revealed ramified morphology of Iba1-positive cells in all investigated groups (Figure 4(b)).

### 3.4. I/R Caused a Significant Increase in the Expression and Activity of MMP9, and Vitamin D3 Normalized This Change

**Figure 5:**
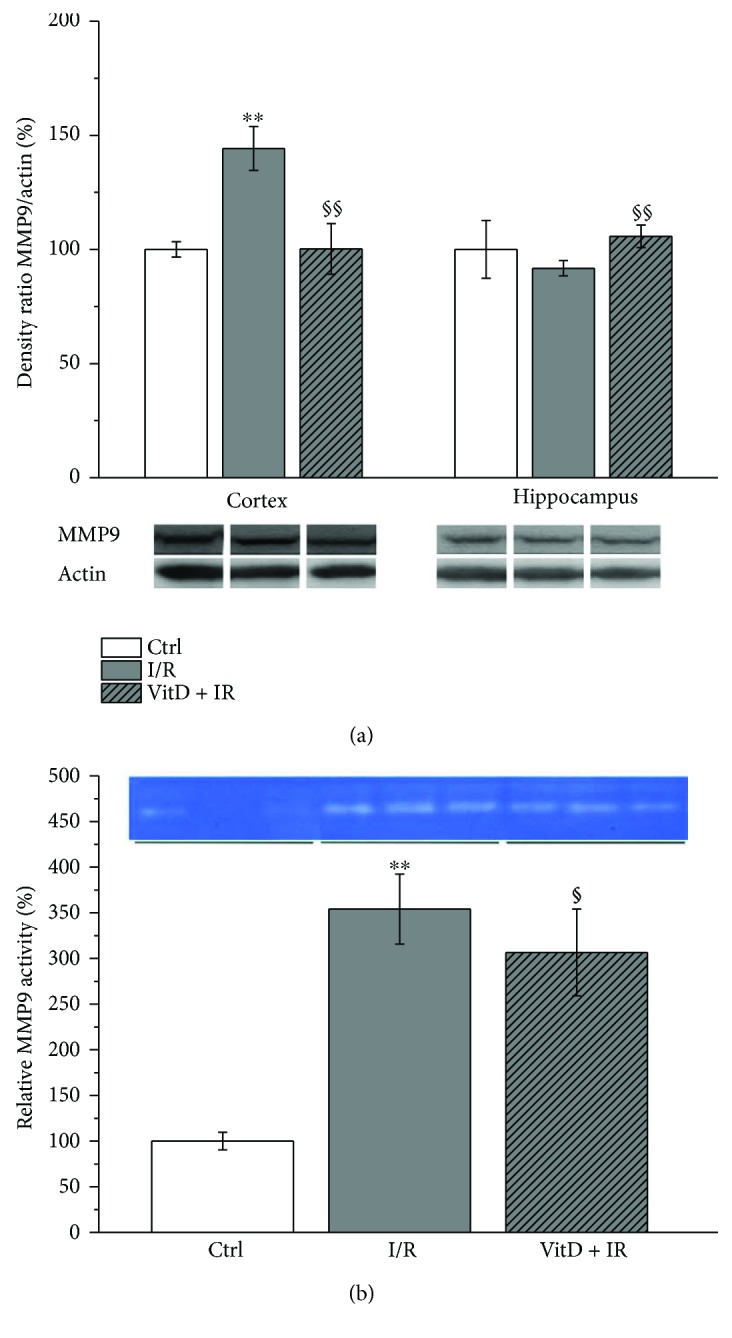
Expression of MMP9. Group I (*n* = 6) was sham-operated animals, which underwent the same surgical procedure without vessel occlusion (control), group II (*n* = 6) was subjected to ischemia/reperfusion (I/R), and group III (*n* = 5) was treated with vitamin D3 and then subjected to I/R (VitD + I/R). All experiments were performed in synaptosomal fraction in different brain structures. (b) Activity of MMP9 in animal serum. Results are presented as mean ± SE. ^∗∗^*p* < 0.01 versus control, ^§^*p* < 0.05 versus I/R, and ^§§^*p* < 0.01 versus I/R.

Changes of the expression of MMP9 were seen only in the cortex. Expression of this protein was increased in the I/R group compared to controls while vitamin D3 attenuated this change (Figure 5(a)).

The activity of MMP9 (Figure 5(b)) in serum was increased in the I/R group compared to control. Vitamin D3 has decreased MMP9 activity in serum with respect to the I/R group.

### 3.5. Expression of VDR

**Figure 6:**
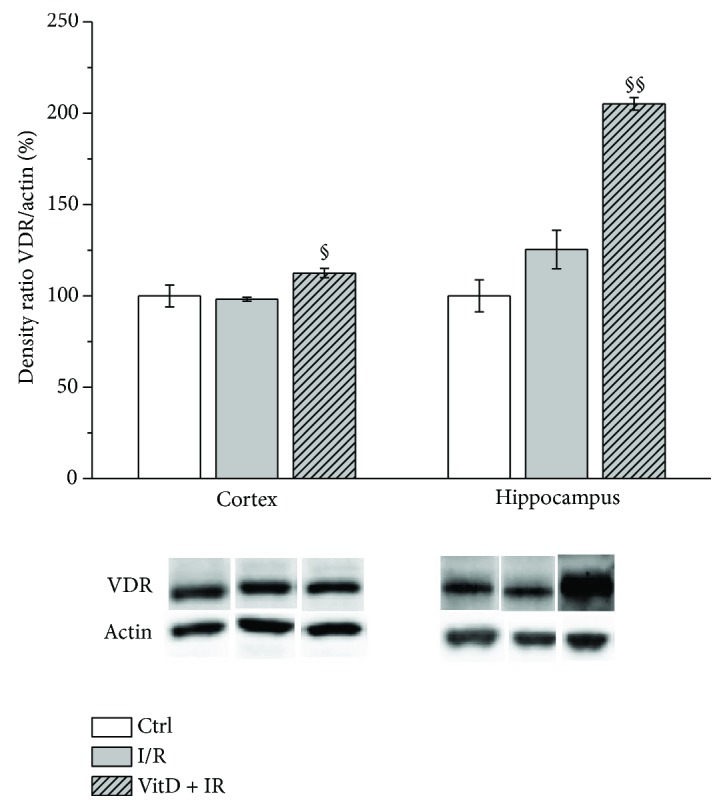
Expression of VDR. Group I (*n* = 6) was sham-operated animals, which underwent the same surgical procedure without vessel occlusion (control), group II (*n* = 6) was subjected to ischemia/reperfusion (I/R), and group III (*n* = 5) was treated with vitamin D3 and then subjected to I/R (VitD + I/R). All experiments were performed in synaptosomal fraction in different brain structures. Results are presented as mean ± SE. ^§^*p* < 0.05 versus I/R and ^§§^*p* < 0.01 versus I/R.

Vitamin D3 supplementation was followed by a significant increase in VDR expression with respect to the I/R group in both structures (Figure 6).

### 3.6. Vitamin D3 Exhibits No Effects on GSH Levels and Enzymes Involved in Its Metabolism

The level of GSH was decreased (Table 1) in all investigated brain structures after I/R; pretreatment with vitamin D3 did not have effects on this change. Among the enzymes involved in glutathione metabolism, the GR activity (Table 2) was increased after I/R and vitamin D3 pretreatment. The activity of GPx and *γ*GCL (Table 2) remained unchanged among experimental groups in the investigated brain structures.

### 3.7. Lipid Peroxidation Expressed by the Levels of MDA Was Increased in the Cortex and Hippocampus after I/R, and Vitamin D3 Pretreatment Has Protective Effects

Measurement of MDA levels (Table 1) revealed an increase in lipid peroxidation in the cortex and hippocampus of the I/R group compared to control, and vitamin D3 reversed these changes.

### 3.8. Superoxide Anion Production Was Increased in All Brain Structures after I/R, and Vitamin D3 Attenuated These Effects

The content of O_2_^·−^ (Table 1) was increased in the investigated brain structures of the I/R group compared to control. Vitamin D3 attenuated these changes completely in the cortex and hippocampus.

### 3.9. Total Activity of SOD Was Increased in the Investigated Brain Structures after I/R

The total activity of SOD (Table 2) was increased in the cortex and hippocampus of the I/R group compared to control. In the VitD + I/R group, the total activity of SOD (Table 2) remained increased with respect to controls, and this increase was statistically significant in the hippocampus.

## 4. Discussion

This study has demonstrated that ten minutes of global transient cerebral ischemia followed by 24 hours of reperfusion results in oxidative stress and changes in the expression of NOX2 that are probably not derived in microglia. Also, the applied I/R procedure caused an increase in MMP9 expression in the brain and activity in the serum. More importantly, this study has demonstrated that vitamin D3 supplementation prior to I/R decreases oxidative stress and NOX2 and MMP9 expressions in the gerbil's brain. Increased expression of the VDR in vitamin D3-pretreated animals subjected to I/R indicates that supplementation is effective even the starting levels of vitamin D.

Although several previous studies have demonstrated the absence of changes in the number and morphology of brain cells 24 hours after brain ischemia [51, 52], subtle biochemical changes that have a potential to be a therapeutic target occur early after ischemia and are present in the first 24 hours of reperfusion [39, 40, 52].

In our study, 10 minutes of global brain ischemia followed by 24 hours of reperfusion did not change the expression of the neuronal marker NeuN and the number or morphology of neurons. However, transmission electron microscopy revealed subtle subcellular changes in the form of accumulation of transport vesicles and rupture of the late endosome membrane in the cells of animals subjected to I/R. These changes were less prominent in the brain of the gerbils treated with vitamin D3 before I/R. Our results are in agreement with the results of the investigation of Cao et al. [51] who have revealed the absence of ischemic lesions one day after 15 minutes of brainstem ischemia in Mongolian gerbils. These authors have found ischemic lesions immediately after ischemia that disappeared after one day and reappeared 3 days after reperfusion. Furthermore, the latest study of Yuan et al. [52] has also demonstrated that 20 minutes of transient global brain ischemia leads to delayed neuronal death that is present after 72 h of reperfusion. These scientists have also shown that although neuron morphology was intact 24 hours after reperfusion, subcellular changes in the form of accumulation of damaged Golgi, transport vesicles, and late endosomes and ribosomes were present.

Several studies have suggested the role of NOX2 as a source of ROS in I/R [5, 53–55]. In our study, the animals subjected to I/R had increased expression of two investigated membrane subunits of NOX2 (gp91^phox^ and p22^phox^) and increased levels of O_2_^·−^ and MDA both in the cortex and in the hippocampus. However, changes of the expression of regulatory cytosolic subunits (p47^phox^ and p67^phox^) differ in the investigated brain structures. In the cortex, p47^phox^ was decreased probably indicating an attempt to establish a balance, while expression of p67^phox^ was not changed. Our results are in accordance with the results of previous studies. Yoshioka et al. [56] have also found upregulation of the membrane subunit gp91^phox^ and the absence of the changes in the expression of regulatory subunits p47^phox^ and p67^phox^ twenty-four hours after 22 minutes of global cerebral ischemia in the striatum of C57BL/6 mice. However, these authors have demonstrated the dynamic of the changes in the expression of cytosolic NOX2 subunits, and upregulation was found in the early (3–6 hours) and late phase (72 hours) after the ischemic procedure [56]. The authors have suggested that NOX activation arose in medium spiny neurons and endothelial cells at the early phase and in reactive microglia at the late phase. Additionally, increased p47^phox^ immunoreactivity was demonstrated in the hippocampus and striatum of the same mice strain 72 hours after 30 minutes of global cerebral ischemia [57]. Supplementation with vitamin D3 prior to ischemia, in our study, was followed by reduced expression of gp91^phox^ and p47^phox^ subunits in both the cortex and hippocampus, increased expression of p22^phox^ in the cortex and decreased expression of this subunit in the hippocampus, and increased expression of p67^phox^ in the hippocampus. Levels of O_2_^·−^ and MDA were decreased as well, suggesting that NOX2 could be an important source of free radicals. The study of Dong et al. [58] has demonstrated reducing effects of vitamin D3 on the expression of NOX2 and NOX4 and the levels of ROS in the models of renovascular hypertension both *in vivo* and *in vitro.* Similarly, Hirata et al. [59] have shown decreased expression of p22^phox^ after the treatment with calcitriol analog 22-oxacalcitriol in an animal model of diabetes mellitus 2, as well as in human coronary artery endothelial cells cultured in high-glucose medium. Vitamin D3 treatment of human promyelocytic NB4 cells has decreased expression of the NOX2 components (p22^phox^, p91^phox^, p47^phox^, and p67^phox^) leading to undetectable levels of gp91^phox^ and p22^phox^ proteins and the low level of p47^phox^ and p67^phox^ [60]. In our study, vitamin D3 has shown structure-specific effects on p22^phox^ and p67^phox^.

Although NOX4 has been implicated as a source of free radicals in transit or permanent focal brain ischemia [4, 14, 61], our study has demonstrated that global transit ischemia lasting for 10 minutes followed by 24 hours of reperfusion was followed with either baseline (cortex) or decreased (hippocampus) expression of NOX4. These results indicate that NOX4 is not an important source of free radicals in this phase of reperfusion. These results are in accordance with the findings of Nishimura et al. [61] who have reported upregulation of NOX4 in brain pericyte culture after exposition to 1% O_2_ hypoxia for over 24 hours and prompted downregulation by reoxygenation with returning to baseline levels within 24 hours. Pretreatment with vitamin D3 in our study kept NOX4 at the baseline level in both investigated brain structures, and this could be of great importance, since it is shown that NOX4 constitutively produces small amounts of H_2_O_2_ affecting vascular dilatation [62]. Additionally, endothelial NOX4 is involved in angiogenesis and subsequent healing process after tissue damage [61]. To the best of our knowledge, there are no results of the effects of global cerebral ischemia on the NOX4. Furthermore, we have found a significant increase in MMP9 expression in the cortex, and this change has been prevented by the pretreatment with vitamin D3. A recent study of Tang et al. [63] has suggested that NOX2 is a major precipitating factor for the increased MMP9 expression in ischemic brain tissue, since 90 min of middle cerebral artery occlusion followed by 22.5 h of reperfusion has increased expressions of both NOX2 and MMP9, while inhibition of NOX2 with apocynin reduced the increase in MMP9. However, we have found increased expressions of both NOX2 and MMP9 in the cortex while in the hippocampus, only NOX2 was elevated. Nishimura et al. [61] have found that NOX4 is responsible for MMP9 activation. Indeed, we have observed a similar expression profile of NOX4 and MMP9 in the hippocampus. It is possible that MMP9 in specific brain regions responds to different signals. The findings of different MMP9 expression in the cortex and hippocampus could also be explained by the dynamic of changes after brain ischemia. It is possible that MMP9 expression would be increased in the hippocampus after a longer period of reperfusion. Besides that NOX influenced the expression of MMPs, it is shown that once-activated microglia release MMPs [64]. Our study has demonstrated ramified morphology of microglia and the absence of changes in the expression of microglial marker Iba1 after the applied I/R procedure. These findings are indicative of the presence of inactivated, resting microglia and suggest other sources of MMP9. In our investigation, MMP9 was also increased in the serum of animals subjected to the I/R procedure and decreased in animals pretreated with vitaminD3. Previous studies have reported that elevated plasma levels of MMP9 correlate with poor neurological outcomes during acute stages of both ischemic stroke and hemorrhagic stroke [19, 23], while Park et al. [20] have demonstrated that the total MMP9 activity in plasma highly correlates with the activity in the brain homogenate. Timms et al. [65] have reported an abnormal increase in circulating MMP9 in subjects with vitamin D3 deficiency. Our study supports the usefulness of MMP9 as a biomarker and indicates the effectiveness of vitamin D3 in reversion of changes of MMP9 caused by I/R. MMPs have been pursued as potential therapeutic targets, and MMP inhibitors were found to be effective in reducing infarct volumes in the animal models of focal cerebral ischemia when administered early during acute ischemic stroke [18]. On the other hand, a biphasic role of MMPs after brain injury has been suggested. In the acute phase, they are found to be deleterious, but in delayed remodeling and recovery, they are potentially beneficial, and blocking MMPs in this phase may even worsen outcomes [18].

Investigations of the antioxidative defense systems in our study have revealed a significant increase in the total SOD activity and a significant decrease in the levels of GSH in investigated brain regions. However, GPx, GR, *γ*GCL activities were unchanged. Previous studies have shown that SOD represents the main endogenous protective system against an excitotoxic and ischemic/hypoxic lesion [66] and that the lower GSH levels are indicative of the greater injury after I/R [39]. Vitamin D3 did not prevent a decrease in GSH caused by the I/R procedure. We have found increased SOD activity in the investigated brain structures in the I/R group compared to control, implying the attempts of antioxidant mechanisms to respond to high oxidative demands. However, the levels of the O_2_^·−^ and MDA were significantly increased with respect to controls. Both findings indicate the presence of oxidative stress. In I/R animals pretreated with vitamin D3, the levels of O_2_^·−^ and MDA were normalized while the activity of SOD was still statistically significantly increased in the hippocampus. These findings indicate protective effects of vitamin D3 against oxidative stress.

Antioxidant capacity of vitamin D3 has been shown in the investigations on the animal model of nonalcoholic fatty liver disease [67]. The authors have applied intraperitoneal injection of 1,25-(OH)_2_ vitamin D3, twice per week, for 16 weeks and have demonstrated protective effects against oxidative stress in the liver. It is suggested that antioxidant capacity of vitamin D3 lays in the induction of NRF2 nuclear translocation factor, upregulation of the expression of genes encoding antioxidant enzymes [67], and inhibition of nuclear factor-kappa B, NOX2, and NOX4 genes [36, 68].

Our findings that vitamin D3 decreases gp91^phox^ and p47^phox^ expression and decreases ROS formation and especially the effects of vitamin D3 supplementation on the increase in the VDR expression support the idea that vitamin D3 supplementation can be recommended to all subjects particularly to those with the high risk for cardiovascular and cerebrovascular disorders, even when they have normal serum levels of 25-OH vitamin D.

Although our investigation has revealed the effects of the I/R procedure and vitamin D3 pretreatment on the NOX2 and NOX4 subunits, MMP9, and parameters of oxidative stress, there are several limitations of the study. First, there is a need to test the dynamic of changes of selected parameters after different durations of ischemia and reperfusion. Moreover, we have tested the effects of vitamin D3 pretreatment in animals that were not vitamin D deficient. It would be necessary to investigate the effects of vitamin D3 supplementation on I/R injury in the group of animals on a vitamin D3-deficient diet. Additionally, treating the animals with vitamin D3 immediately after the brain ischemic procedure would provide an insight into wholesomeness of vitamin D3 application early after cerebrovascular incidents. Finally, the different protocols and given doses should be tested.

## 5. Conclusions

Ten minutes of global cerebral ischemia followed by 24 hours of reperfusion induces changes in the expression of NOX2 subunits and MMP9 and changes of the oxidative stress parameters in the brain, as well as the increase in MMP9 activity in the serum of experimental animals. These results emphasize the role of NOX2 and MMP9 in the pathophysiology of brain ischemia. Pretreatment with vitamin D3, followed by the increase in VDR expression, is especially effective on the changes of the membrane NOX2 subunits, MMP9, and the levels of MDA and O_2_^·^−. We are stressing the significance of vitamin D3 supplementation in ameliorating the injury caused by ischemia/reperfusion.

## Figures and Tables

**Table 1:** Levels of GSH, MDA, and O_2_^·−^ in the synaptosomal fraction of different brain structures in group I (*n* = 6) (sham-operated animals, which underwent the same surgical procedure without vessel occlusion (control)), group II (*n* = 6) (subjected to ischemia/reperfusion (I/R)), and group III (*n* = 5) (treated with vitamin D3 prior to I/R (VitD3 + I/R).

Brain structure		Cortex	Hippocampus
Parameter	Group		
GSH (mmol/mg protein)	Ctrl	12.77 ± 0.59	12.06 ± 0.90
	I/R	7.07 ± 1.03^∗^	8.03 ± 1.14^∗^
	VitD3 + I/R	4.13 ± 0.62^∗§^	6.80 ± 1.20^∗^

MDA (mmol/mg protein)	Ctrl	35.22 ± 1.63	29.68 ± 0.64
	I/R	51.33 ± 5.73^∗^	38.24 ± 2.61
	VitD3 + I/R	39.51 ± 4.19^§^	32.19 ± 2.77^§^

O_2_^·−^ (nM/min/mg protein)	Ctrl	8.09 ± 0.34	9.82 ± 0.25
	I/R	10.88 ± 0.29^∗∗^	11.47 ± 0.34^∗∗^
	VitD3 + I/R	8.93 ± 0.33^§§^	10.19 ± 0.28^§§^

Results are presented as mean ± SE. ^∗^*p* < 0.05 versus control, ^∗∗^*p* < 0.01 versus control, ^§^*p* < 0.05 versus I/R, and ^§§^*p* < 0.01 versus I/R.

**Table 2:** Activities of the GPx, GR, *γ*GCL, and total SOD in the synaptosomal fraction of different brain structures in Group I (*n* = 6) (sham-operated animals, which underwent the same surgical procedure without vessel occlusion (control)), group II (*n* = 6) (subjected to ischemia/reperfusion (I/R)), and group III (*n* = 5) (treated with vitamin D3 prior to I/R (VitD3 + I/R).

Brain structure		Cortex	Hippocampus
Parameter	Group		
GPx (U/mg protein)	Ctrl	29.34 ± 4.20	38.91 ± 3.62
	I/R	31.40 ± 3.91	27.85 ± 5.22
	VitD + I/R	31.60 ± 1.78	26.25 ± 4.79

GR (U/mg protein)	Ctrl	15.76 ± 3.1	6.68 ± 1.1
	I/R	28.58 ± 5.11^∗^	28.43 ± 5.26^∗∗^
	VitD + I/R	38.19 ± 4.11	19.43 ± 4.83

*γ*GCL (U/mg protein)	Ctrl	15.76 ± 0.11	6.68 ± 0.43
	I/R	28.58 ± 0.10^∗^	28.43 ± 0.12^∗^
	VitD + I/R	38.19 ± 0.08^∗^	19.43 ± 0.29

SOD (U/mg protein)	Ctrl	128.61 ± 3.11	129.52 ± 1.11
	I/R	183.36 ± 5.11^∗∗^	192.26 ± 5.26^∗∗^
	VitD + I/R	145.04 ± 4.05	184.37 ± 4.83^∗^

Results are presented as mean ± SE. ^∗^*p* < 0.05 versus control and ^∗∗^*p* < 0.01 versus control.

## Abbreviations

ADP: Adenosine diphosphate
ATP: Adenosine triphosphate
BBB: Blood-brain barrier
BCAO: Both common carotid artery occlusion
CNS: Central nervous system
DTNB: Dithionitrobenzoic acid
DUOX 2: Dual oxidase 2
GD: Gyrus dentatus
*γ*GCL: Gamma-glutamylcysteine ligase
GPx: Glutathione peroxidase
GR: Glutathione reductase
GSH: Glutathione
Iba1: Ionized calcium-binding adapter molecule 1
I/R: Ischemia reperfusion
MDA: Malondialdehyde
MMP: Matrix metalloproteinases
NADH: Nicotine adenine dinucleotide
NADPH: Nicotine adenine dinucleotide phosphate
NOS: Nitric oxide synthase
NOX: NADPH oxidase
RSA: Retrosplenial agranular cortex
RSG: Retrosplenial granular cortex
ROS: Reactive oxygen species
SDS: Sodium dodecyl sulfate
SDS-PAGE: Sodium dodecyl sulfate polyacrylamide gel electrophoresis
SOD: Superoxide dismutase
SOD1: Copper-zinc superoxide dismutase
SOD2: Manganese superoxide dismutase
TBST: Tris-buffered solution/Tween 20
TIMP: Tissue inhibitor of metalloproteinase
VitD3: Vitamin D3 (1,25-dihydroxycholecalciferol).
